# Resolving Electron-Transfer Heterogeneity in Electrochemical Aptamer-Based Sensors by Distribution of Capacitive Times

**DOI:** 10.1149/1945-7111/aeb765

**Published:** 2026-11-12

**Authors:** Bob M. Lansdorp, Jennifer M. Gibson, Lior Sepunaru, Kevin W. Plaxco

**Affiliations:** 1 Hawai‘i Natural Energy Institute, University of Hawai‘i at Mānoa, Honolulu, HI, 96822, United States of America; 2 Interdisciplinary Program in Quantitative Biosciences, University of California, Santa Barbara, CA 93106, United States of America; 3 Department of Chemistry and Biochemistry, University of California, Santa Barbara, CA 93106, United States of America; 4 Department of BioEngineering, University of California, Santa Barbara, CA, 93106, United States of America

## Abstract

Distribution of timescales methods have become powerful tools for extracting hidden kinetic processes from electrochemical impedance spectra. Their physical interpretation, however, depends critically on matching the assumed mathematical model to the underlying physical electrochemical system. Here, we show that the Distribution of Capacitive Times (DCT) model is well-matched to the physics of surface-immobilized redox systems, enabling electron-transfer rate heterogeneity to be resolved. This makes electrochemical aptamer-based (EAB) sensors a natural system for DCT analysis, as their surface-tethered redox reporters can occupy distinct molecular states with different electron-transfer kinetics. Applying DCT to a vancomycin EAB biosensor reveals two distinct electron-transfer populations whose respective rates remain nearly constant as a function of vancomycin concentration. In contrast, their relative occupancies evolve with changing target concentration, demonstrating that the sensor’s response arises from redistribution between states rather than from a continuously varying electron-transfer rate. Analysis of the population amplitudes is consistent with a biophysical model in which approximately one-third of the surface-bound aptamers do not respond to target, suggesting an area that can be explored to improve the performance of such sensors. These results demonstrate that DCT analysis can uncover mechanistic insights in surface-immobilized redox systems that are inaccessible to conventional impedance analysis.

## Highlights

Advancing the Distribution of Capacitive Times (DCT) method by isolating solution resistance and restricting the frequency range
Demonstrating that vancomycin binding shifts molecules between two electron-transfer-rate states in an electrochemical aptamer-based (EAB) sensor
Analyzing the aptamer folded fraction as a function of concentration to resolve a non-folding state.

## Graphical abstract

**Figure:**
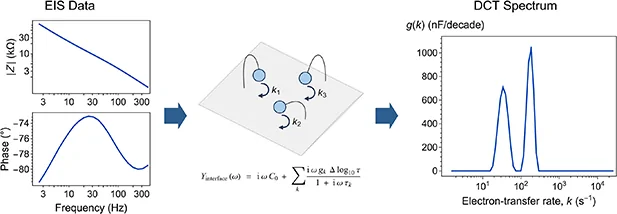

Electrochemical systems are often heterogeneous in multiple dimensions. The electrode/electrolyte interface that separates the electron-conductive solid from the ion-conductive liquid, for example, is so ubiquitous that heterogeneous electron transfer is often defined as the charge transfer in a direction normal to the solid/liquid interface.^1^ However, heterogeneity in the electron transfer rate from site to site along the solid/liquid interface is also possible, and could arise from a wide range of sources^2^ including variations in electrode morphology, molecular coverage,^3^ intermolecular interactions, tether lengths,^4^ tilt angles, re-organization energy variation, and different molecular conformational states.^5^ In systems such as electrochemical biosensors, understanding and resolving the distribution in electron transfer rates is essential for distinguishing the physicochemical processes that contribute to the measured electrochemical response, and thus to their performance.

One widely used method for probing interfacial processes in electrochemical systems is Electrochemical impedance spectroscopy (EIS).^6^^–^^8^ A typical EIS measurement involves applying small sinusoidal voltages over a wide range of frequencies and recording the current to generate impedance spectra. A commonly used technique to interpret EIS spectra is the Distribution of Relaxation Times (DRT) method, which has produced important insights in batteries, fuel cells, and solid-state ionic conductors.^9^^–^^11^ As has been previously described:^12^*“The Distribution of Relaxation Times (as is currently employed) is a method to express electrochemical impedance spectra as a function of serially connected Voigt elements over a continuous set of time constants expressed through basis functions.”* The aforementioned Voigt elements, which consist of parallel resistors and capacitors connected in series, are well-suited to describe electrochemical systems in which current flows sequentially through material domains, each with their own timescale-dependent charge-transfer resistance. Many electrochemical systems, however, do not conform to the Voigt element ladder assumption inherent in DRT. Electrodes functionalized with covalently attached redox reporters, for example, are characterized by heterogeneous electron transfer across an otherwise insulating solid/liquid interface. This insulating layer, also known as a blocking layer, creates an infinite impedance at low frequency, which violates the assumptions inherent to DRT.^13^ Given this, an alternative approach, the “Distribution of Capacitive Times” (DCT) method has been introduced,^13^^,^^14^ which removes the Voigt ladder in favor of a Maxwell ladder, thus replacing the series-connected elements with parallel-connected elements (Fig. 1). The technique yields the same results as the Distribution of Differential Capacity,^14^ which divides the admittance by iω and fits to that quantity instead of, as we do here, fitting in admittance space to obtain the DCT spectrum.

**Figure 1.**
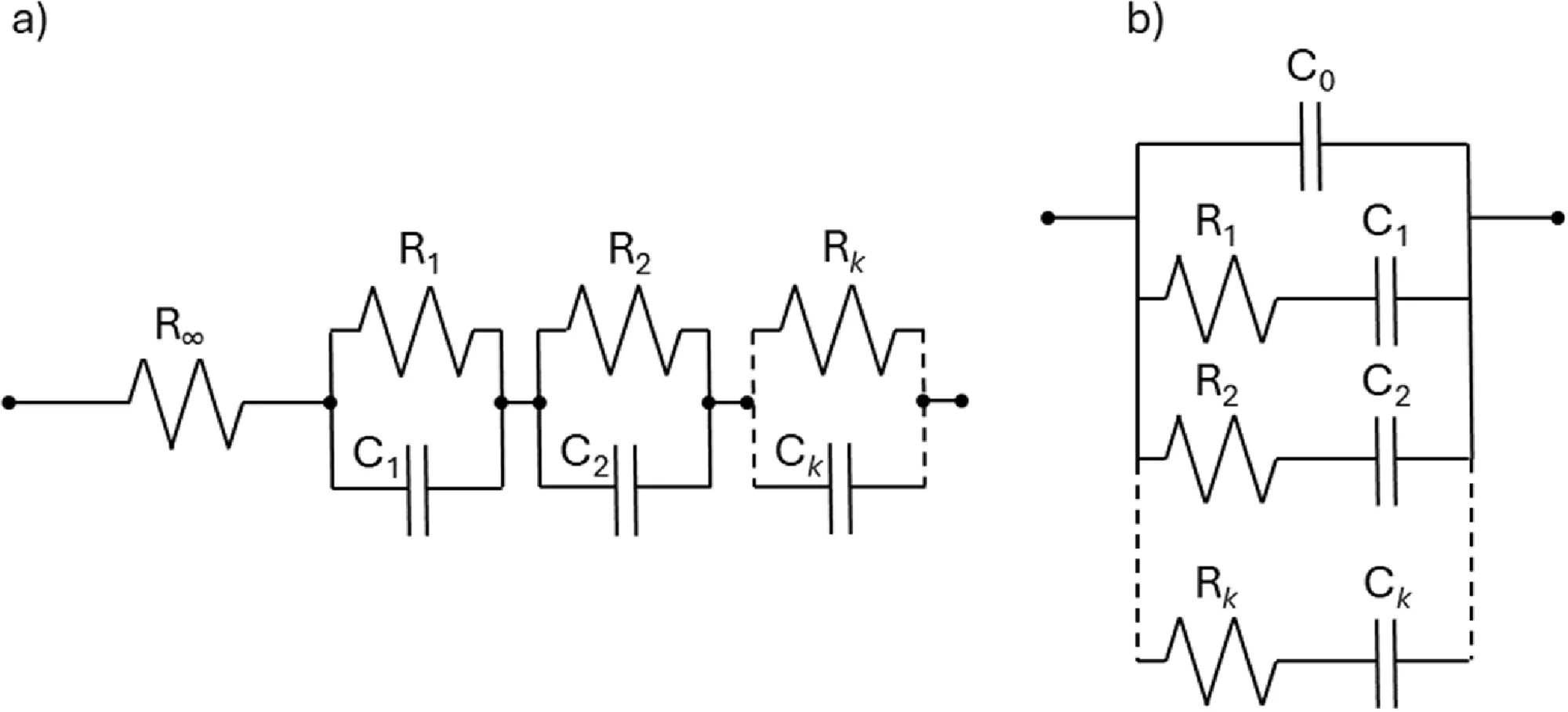
The choice of equivalent circuit encodes a physical assumption about how current flows through the system. (a) The Voigt ladder underlying the Distribution of Relaxation Times (DRT) method places resistor-capacitor pairs in series, appropriate when current passes sequentially through stacked material domains. (b) The Maxwell ladder underlying the Distribution of Capacitive Times (DCT) method places them in parallel, the natural description of surface-immobilized redox molecules sharing a single insulating solid/liquid interface.

Within the EIS fitting community, there appears to be a polarization, with one side advancing new mathematical tools with the goal of making highly generalizable analysis methods,^11^^,^^13^^,^^15^^,^^16^ and the other side pointing out that philosophically it is impossible to make a fully generalizable EIS analysis method.^12^ We thus identify a gap in the scientific literature: is there a middle ground where justifiable assumptions can be used to make useful advancements to our understanding? Unlike solution-phase redox systems, surface-immobilized redox molecules do not undergo translational diffusion and lack a Warburg contribution (see Ref. 1, Section 17.3.3). Consequently, their interfacial response can be modeled as a superposition of linear circuit elements. Specifically, since the redox-active molecules are covalently linked to a self-assembled monolayer, they can be modeled as parallel circuit elements, each with a charge transfer resistance and pseudo-capacitance. Thus, for surface-immobilized redox reporters which are covalently attached to the surface, DCT is a natural choice for a model, and as we will show, proceeding with this model leads to advancements in our understanding of the underlying physics.

## DCT theory.

The DCT workflow (Fig. 2) begins with the acquisition of an EIS spectrum, which can be represented in Bode amplitude and phase plots (Fig. 2a). In the Maxwell ladder representation (Fig. 2b), each resistor-capacitor element corresponds to a population of molecules characterized by a particular electron-transfer timescale, *τ*_1_, *τ*_2_, *τ*_3_, etc. Faster and slower populations contribute independently to the total admittance, which is simply the sum of all parallel contributions together with a frequency-independent capacitance. Finally, mathematical inversion of the measured admittance yields a distribution of capacitance timescales, *g*(*k*), which quantifies the capacitance associated with each electron-transfer rate (Fig. 2c). We note that, in contrast to the Distribution of Relaxation Times (DRT) method, which yields resistance density (ohms/decade), the ${g_k}$ in DCT yields capacitance density (farads/decade), with distinctly transferring populations of surface-bound molecules appearing as separate peaks in the resulting DCT spectrum.

**Figure 2.**
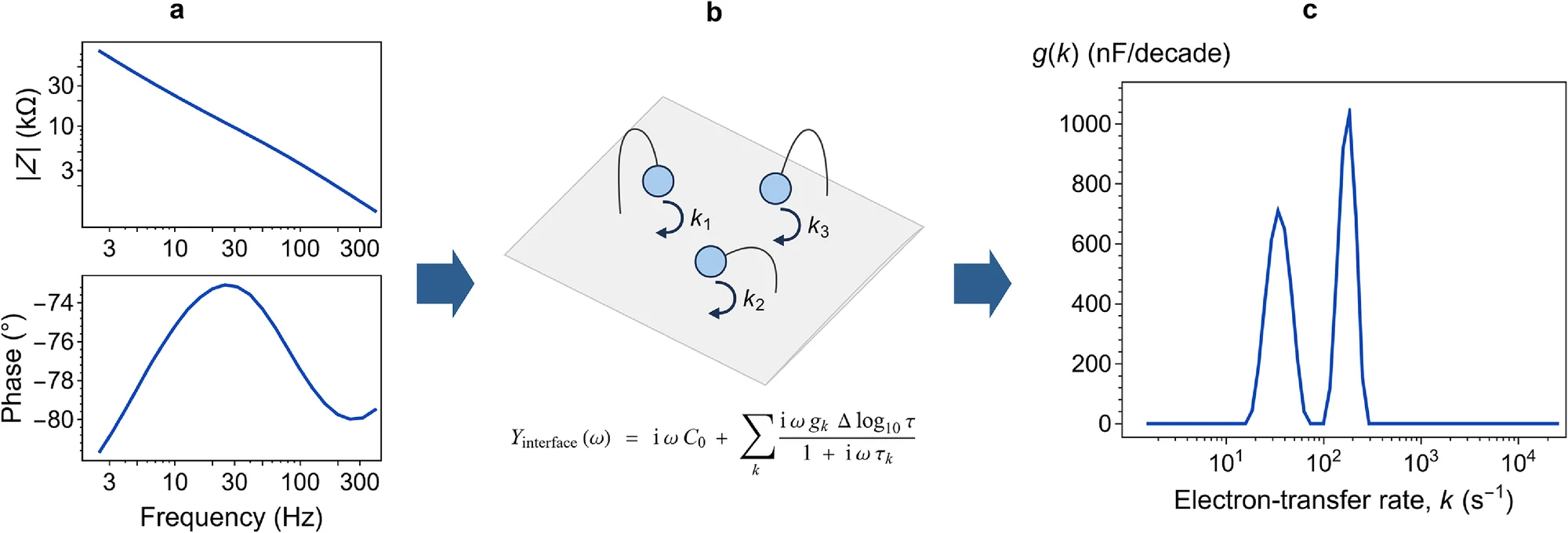
DCT converts a frequency-domain measurement into a distribution of electron-transfer rates. An EIS spectrum (a) undergoes mathematical inversion (b) to yield a DCT spectrum (c), in which each distinct electron-transfer population appears as a separate peak. Representative data shown is from Electrode B1 measured at 178 μM of vancomycin.

Below, we will derive the admittance function for a surface covered with molecules, each with its own electron transfer rate, which will be used in subsequent fitting to experimentally measured admittance data. Each redox-active molecule can be thought of as a single charge-transfer resistor ${R_i}$ in series with a pseudo-capacitance ${C_i}$, connected in parallel across the interface. A large number of these molecules make up a Maxwell ladder, with the impedance of each element in the ladder being given by: ${Z_i}(\omega ) = {R_i}+\frac 1{i\omega {C_i}}$. Substituting ${\tau_i} = {R_i}{{\ C}_i}$ we obtain the admittance ${Y_i}(\omega ) = \frac{i\omega {C_i}}{1+i\omega {\tau_i}}$, which reveals the underlying Maxwell kernel:

\begin{equation*}{K_{\mathrm{Maxwell}}}\left(\omega ,\tau \right) = \frac{i\omega}{1+i\omega {\tau_i}}.\end{equation*}

Because parallel admittances sum, after adding a frequency independent capacitance component ${C_0}$ the net admittance of the ladder is:

\begin{equation*}{Y_{\mathrm{net}}}\left(\omega \right) = i\omega {C_0}+\sum \limits_{i = 1}^{N}\frac{i\omega {C_i}}{1+i\omega {\tau_i}}.\end{equation*}

Next, we assume that the interface contains sufficiently many redox active species and sufficient heterogeneity that the system can be well-described by a continuous distribution of smoothly varying capacitance timescales. To span the orders of magnitude of timescales experimentally accessible by EIS, we construct a discrete ladder of timescales ${\tau_k}$ spaced evenly on a logarithmic grid with ${\tau _{\min }} = \frac{1}{{2\pi {f_{\max }}}}$ and ${\tau _{\max }} = \frac{1}{{2\pi {f_{\min }}}}$. We then define g(τ) as the capacitance density with respect to logarithmic timescale, such that the area under the curve of the g(τ) versus ${\log _{10}}\left( \tau \right)$ corresponds to the total capacitance in the Maxwell ladder. Throughout this work we make the substitution $k = \frac 1{\tau}$. Because *g* is defined per decade and a decade in *τ* is a decade in *k* (i.e. |d log_10_*k*| = |d log_10_*τ*|), the substitution leaves the distribution unchanged: *g*(*k*) equals *g*(*τ*) evaluated at *τ* = 1/*k*, with the axis reversed. In contrast to charge transfer resistance, which is an extrinsic parameter that depends on both electron transfer rate and the total number of aptamers, the shape of the g(τ) spectrum (i.e. the peaks and their locations) represents an intrinsic signature of the molecular kinetics that can be more readily compared across measurements. It is possible to convert the amplitude of g(τ) into estimates of molecular surface density using additional knowledge about the system (appendix), but in the analysis below we avoid the conversion in order to invoke the minimum number of assumptions, and report values in terms of capacitance rather than surface density. The interfacial admittance model can then be written using a discrete approximation to the continuous function with elements ${g_k}$:

\begin{equation*}{Y_{\mathrm{interface}}}\left(\omega \right) = i\omega {C_0}+\sum \nolimits_k \frac{i\omega {g_k}\,\,{\Delta} {\log_{10}}\tau}{1+i\omega {\tau_k}}\end{equation*}

where $\Delta {\log _{10}}\tau = $ 1/(bins per decade) is the width of each logarithmically-spaced bin. We note that Eq. (2) is a sum over all molecules on the interface, which is typically an impractically large number to compute. In contrast, Eq. (3) is a discrete sum over all bins in the approximation to the continuum limit. To facilitate comparison between the theoretical model captured by Eq. (3) and actual, experimentally-obtained EIS data, the former is typically binned at the same density that the latter are collected at, which is typically around 10 bins per decade.

## Experimental

**Sensor fabrication.** Gold wire working electrodes (200 µm diameter, 99.9% purity; ThermoFisher Scientific) were cut to 4.5 cm and soldered to gold-plated pin connectors (CH Instruments, Inc) with 60/40 lead-tin solder (DigiKey Electronics). Polyolefin heat-shrink tubing (0.356 mm expanded inner diameter; McMaster-Carr) was applied with a heat gun (80 °C) to insulate 3.4 cm of each wire, leaving a 3 mm exposed gold segment as the working length. A modified thermoplastic liquid connector coating (DigiKey Electronics) was applied at the wire-pin junction for additional insulation.

As our primary testbed, we employed electrochemical aptamer-based (EAB) sensors functionalized with a 4-base truncated variant of a previously reported vancomycin-binding aptamer (5′-SS-(CH_2_)_6_-CGA GGG TAC CGC AAT AGT ACT TAT TGT TCG CCT ATT GTG GGT CGG-methylene blue-3′, Integrated DNA Technologies). Lyophilized DNA was resuspended in ultrapure water to ∼100 µM and stored in 2 µl aliquots at −20 °C. Immediately before use, a thawed aliquot was combined with 14 µl of 10 mM TCEP·HCl (MilliporeSigma, in ultrapure water) and incubated for 1 h at room temperature in the dark to reduce the 5′ disulfide. The reduced aptamer was then diluted to 500 nM in 1 × PBS (pH 7.4; Fisher Scientific), prepared from a 20 × PBS stock (Santa Cruz Biotechnology) in ultrapure water.

Prior to functionalization, the exposed gold surface was electrochemically cleaned using an external platinum rod counter electrode and aqueous Ag/AgCl reference electrode (both CH Instruments, Inc.). Organic contaminants were removed first by cycling the gold working electrode potential between −1 V and −2 V (all reported potentials are versus Ag/AgCl) at 1 V s^−1^ in 0.5 M NaOH (MilliporeSigma) for 1000 cycles, followed by cycling between 0 V and +1.8 V at 1 V s^−1^ in 0.5 M H_2_SO_4_ (MilliporeSigma) for 100 cycles to strip residual inorganic and organic material. The electroactive surface area was then increased by electrochemical roughening in 0.5 M H_2_SO_4_, using chronoamperometric potential steps between 0 V and +2.2 V repeated 100 times, as previously described.^17^ Cleaned electrodes were incubated in 500 nM reduced aptamer (1 × PBS) for 1 h at room temperature in the dark, then transferred to 10 mM 6-mercapto-1-hexanol (MCH; MilliporeSigma) in the same buffer and left overnight at room temperature in the dark to backfill the gold surface and form a self-assembled monolayer. Electrodes were rinsed with ultrapure water immediately before each measurement.

**Electrochemical measurements.** Cyclic voltammetry and electrochemical impedance spectroscopy (EIS) were performed using an Autolab PGStat128N (Metrohm). Cyclic voltammetry scans were collected at the start of each experiment, recorded in staircase mode (NOVA “CV staircase”): the potential was stepped from 0 V to +0.01 V, down to −0.45 V and back to 0 V versus the reference electrode in 2.44 mV increments, each held for 24.4 ms, with the current recorded once per step (see Fig. S1).

EIS spectra were acquired from 10 000 Hz to 0.1 Hz (10 frequencies per decade) at a 10 mV root-mean-squared amplitude, with the DC bias set to the redox potential of the methylene blue reporter as determined from the corresponding staircase cyclic voltammogram.

Concentration titrations were performed on two independent sets of three electrodes on separate days, labeled Titration A (electrodes A1–A3; 0, 100, 145, 200, and 250 µM) and Titration B (electrodes B1–B3; 0, 1, 10, 100, 178, 316, 562, and 1000 µM). The full measurement schedule is given in Table S3. In each titration, target was added incrementally in ascending order: at each step, a volume of solution equal to the aliquot to be added was first withdrawn from the electrochemical cell, and the same volume of vancomycin stock was then added, keeping the cell volume constant. The solution was mixed by pipetting up and down 10 times and allowed to equilibrate for 1 min before measurement. One EIS spectrum was collected per concentration (Fig. S2). The drift experiment was performed over 24 h after the second titration concluded (Fig. S3).

### DCT numerical implementation.

In most practical electrochemical experiments, a solution resistance *R*_s_ is present in series with the surface interface.^1^ The presence of this resistance complicates the resulting mathematics of the DCT method and associated fitting, which assumes only additive admittance on the interface. Given this, it was previously anticipated^18^ that *“it might be simpler to fit the admittance spectrum after subtracting the contribution from the top resistor and capacitor, in a way analogous to how impedance is fitted for a Voight [sic] configuration.”* In order to compute both the solution resistance and the Maxwell ladder elements simultaneously, we use an outer loop to obtain ${R_\mathrm{s}}$ and an inner loop on ${Y_{\mathrm{interface}}}$. The outer loop for ${R_s}$ simply minimizes the square error of admittance. For each iteration of the inner loop, the interfacial admittance is calculated by subtracting the solution resistance from the raw EIS data ${Z_{\mathrm{data}}}(\omega )$ and inverting:

\begin{equation*}{Y_{\mathrm{interface}}}\left(\omega \right) = \frac 1{{Z_{\mathrm{data}}}\left(\omega \right)-{R_\mathrm{s}}}.\end{equation*}

The advantage of fitting the solution resistance in a separate loop to the Maxwell ladder is that the inner loop ${Y_{\mathrm{interface}}}$ is linear in the coefficients ${g_k}$, allowing us to write:

\begin{equation*}{Y_{\mathit{interface}}} = A\text{ }\cdot \text{ }x\text{, with}\end{equation*}

\begin{equation*}{A_{j,k}} = \left\{ {\begin{array}{*{20}{c}} {i{\omega _j},}&amp;{k = 0} \\[7pt] {\frac{{i{\omega _j}\;\Delta {{\log }_{10}}\tau }}{{1 + i{\omega _j}{\tau _k}}},}&amp;{k \ge 1} \end{array}} \right.\end{equation*}

where $x = [{C_0},{g_1},\ldots ,{g_N}]$ is the vector of coefficients to solve for.

While a neural network approach has previously been used to solve the DCT equations,^13^ well-established methods of linear algebra can also solve them,^14^ which is the approach we have taken here. Moreover, using linear algebra, as opposed to non-linear fitting or neural networks, has the added advantage that the resulting solution is guaranteed to solve the Maxwell ladder coefficients with the global minimum error, which adds confidence that the resulting DCT spectra are not just a local minimum that is dependent on the initial estimates. The non-negative-least-squares formulation guarantees convergence to a global minimum of the least-squares objective, but this minimum is not necessarily unique. Because the forward operator is strongly smoothing in timescale space, multiple distinct distributions can yield nearly identical residual error; regularization is therefore required to obtain reliable DCT spectra.

### Weighting and regularization.

The ideal least squares fits each data point with a weight inversely proportional to its variance. We found empirically that our EIS data exhibited a variance of admittance roughly proportional to mean admittance to the power of a constant *P* (see Fig. S4). Thus, we scale each data point using the weight:

\begin{equation*}W\left(\omega \right) = {|{Y_{\mathrm{interface}}}\left(\omega \right)|^{-P}}.\end{equation*}

In order to balance data misfit and solution roughness, we apply the additional assumption that the DCT spectrum g(τ) is smoothly varying in τ. This assumption allows us to apply Tikhonov regularization^19^ by penalizing the second finite difference of the distribution *g_k_*. The strength of regularization is $\lambda = {\lambda_{\mathrm{nd}}}\ {({s_A})^2}$, where ${\lambda_{\mathrm{nd}}}$ is a user-specified dimensionless value (e.g. ${\lambda_{\mathrm{nd}}} = 1{0^{-3}}$), and ${({s_A})^2}$ is the median squared norm of the columns in the design matrix (excluding the *C*_0_ term). This allows us to choose a non-dimensional regularization parameter ${\lambda_{\mathrm{nd}}}$ that can be applied across different data sets with different admittance magnitudes. We use Tikhonov regularization to balance two competing failure modes. Too little regularization (small ${\lambda_{\mathrm{nd}}}$) yields spectra that are excessively spiked, introducing spurious peaks from fitting measurement noise. In contrast, too much regularization (large ${\lambda_{\mathrm{nd}}}$) over-smooths the distribution, merging physically distinct peaks into a single broad feature. We selected ${\lambda_{\mathrm{nd}}}$ = 10^−3^ to balance these extremes, suppressing noise-driven artifacts while retaining the resolution needed to distinguish genuine electron-transfer populations.

The admittance equations are converted into a real-valued system by concatenating the real and imaginary parts. The final objective minimized is:

\begin{equation*}{\left| \left| {W^{\frac 1{2}}}\left(A\ x-b\right)\right| \right| ^2}+\lambda {||D\ x||^2}\end{equation*}

where W is the diagonal matrix of admittance weights, A is the kernel matrix, *x* = [*C*_0_, *g*_1_, …, *g_N_*] are coefficients to solve, $b = {Y_{\mathrm{interface}}}$ are experimentally measured values of interfacial admittance, λ is the regularization parameter, and *D* is a second-difference matrix that applies only to the distribution components. This inner loop admittance equation is solved using Non-Negative Least Squares using the Lawson and Hanson active-set algorithm,^20^ implemented in Mathematica following the public-domain code of M. Woodhams (2003). The algorithm imposes positivity constraints (x≥0) on the terms, while the outer loop finds the best fit *R*_s_ using scalar minimization in admittance space. The code to reproduce these results is accessible at github.com/boblansdorp/DCT.

### Validation on simulated data.

A first test for the linear-algebra based DCT algorithm we have developed is to simulate EIS data and see how closely we can extract the original spectrum used to generate them. To do so we used a DCT spectrum which consisted of a frequency comb, with peaks at 10 s^−1^, 100 s^−1^, and 1000 s^−1^ in parallel with a 100 nF capacitor and with a 50 Ω resistor in series in order to generate simulated EIS data with 15 points per decade. We extracted the resistance, capacitance, areas, and mean electron transfer rates of all three peaks from simulated EIS data using DCT, and found that all were within 1% error of the original simulated parameters (Fig. S5, Table S1), demonstrating that our DCT implementation is functioning as intended and is ready to be applied to real data.

### Selection of frequency range and weighting from measured data.

In order to maximize the goodness of fit between the kernel and system, we restricted the frequency range over which DCT fitting was performed as compared to the raw data, which was collected between 0.1 Hz to 10 kHz. We used the Kramers–Kronig relations, which hold only when the system is stable and time-invariant during the measurement, to assess data quality across our frequency range.^21^ We used raw data and did not subtract trends, but in selecting the median value for which the unweighted Kramers–Kronig error deviated by 2%, we obtained a minimum frequency for subsequent fits of ${f_{\min }} = 2.512$ Hz and discarded the lower frequency data (Fig. S6). This choice to discard low frequency data was further supported by our observation that the phase below 2.512 Hz did not asymptote to 90° as would be expected for a purely blocking layer. We plotted the variance of measured admittance against mean admittance and found that the behavior of the variance exhibited a dip above 500 Hz, which we speculate may be due to the potentiostat control system interacting with the system impedance in this frequency range to yield non-physical readings, motivating our selection of ${f_{\max}} = 500 \text{ } \mathrm{Hz}$, which in practice was equal to ${f_{\max}}$ = 398 Hz since that was the highest frequency measured below 500 Hz. Between ${f_{\min}}$ and ${f_{\max}}$, we fit the variance vs mean admittance to a power law to obtain the exponent P of 0.87 (Fig. S4) which we used to weight data inverse to variance in subsequent fitting. We chose a regularization parameter of ${\lambda_{\mathrm{nd}}}$ = 10^−3^ to balance data misfit and solution roughness (Figs. S7 and S8), and 15 points per decade in the DCT spectrum. We fit the resulting EIS spectra with a DCT model over a frequency range of ${f_{\min}}$–${f_{\max}}$ (Fig. S9), corresponding to electron transfer rates which are a factor of 2π higher than EIS frequencies of 1.578 × 10^1^ s^−1^ and 3.1 × 10^3^ s^−1^ respectively, which we further padded by an order of magnitude on either side in order to minimize edge effects, corresponding to a DCT spectrum electron transfer rate range of ∼1.6 s^−1^ up to ∼3.1 × 10^4^ s^−1^.

## Results

### DCT analysis of a vancomycin electrochemical aptamer-based biosensor.

As our test-bed, we used an electrochemical aptamer-based (EAB) sensor against the antibiotic vancomycin.^22^ EAB sensors use a redox reporter attached to a DNA-based molecular recognition sequence (an aptamer) to measure the concentration of specific molecular analytes. Since their initial introduction^23^ these systems have been well characterized,^7^^,^^22^^,^^24^^–^^35^ and are beginning to see transition toward the clinic.^36^ With their increasing ubiquity, there is an increasing need to understand the fundamental mechanisms underlying EAB sensor operation^37^ through new methodological developments.^38^ To this end, we have previously employed Lovrić plots to monitor the binding-induced change in electron transfer kinetics seen in EAB sensors.^39^ Such studies, however, are ultimately limited by the inability of Lovrić plots to yield distinctly resolved peaks. In response, here we show that DCT can provide high-resolution estimates of the electron transfer kinetics of discrete EAB sensor states, thus revealing insights into their underlying biophysics.

Our first tests involved measuring EAB sensors using staircase cyclic voltammetry to determine the peak potential for subsequent EIS interrogation (Fig. S1). Similar to square wave voltammetry, cyclic voltammetry does not have sufficient fidelity to resolve multiple states with similar electron transfer rates. We then measured EAB sensors over 24 h in a simple buffer at a constant vancomycin concentration, conditions under which such sensors exhibit only a relatively small change in measured admittance over repeated interrogations. This allowed us to optimize the frequency range and weighting parameters for DCT fitting (see Selection of frequency range and weighting from measured data). Using the above parameters, we obtained estimates for the solution resistance and double-layer capacitance as a function of time (Fig. S10). More significantly, we obtained the first DCT spectra of an EAB sensor (Figs. 3 and S11). The presence of well-defined peaks in the DCT spectra provides an initial indication that the assumed Maxwell kernel provides a good match to the physics of EAB sensors, since mismatched kernels typically cause broad peaks spanning many orders of magnitude in frequency. That said, the presence of sharp peaks alone is insufficient evidence that they correspond to real electrochemical processes. If we further compare the reconstructed impedance using this DCT spectrum with the raw measured EIS spectra, we find less than 4% amplitude error and 3° of phase error over the entire fit frequency range, strengthening the evidence of a good fit between the assumed kernel and the measured system (Fig. S12). We find that the two peaks remain prominent across different weighting exponents (Fig. S13). Finally, the two peaks decreased by only 21% in amplitude over 24 h of interrogation, which is aligned with previous reports of surface loss of aptamers after repeated interrogation,^39^^–^^41^ and in particular measurements performed using electrochemical impedance spectroscopy with a narrower potential range than traditional square wave voltammetry.^8^

**Figure 3.**
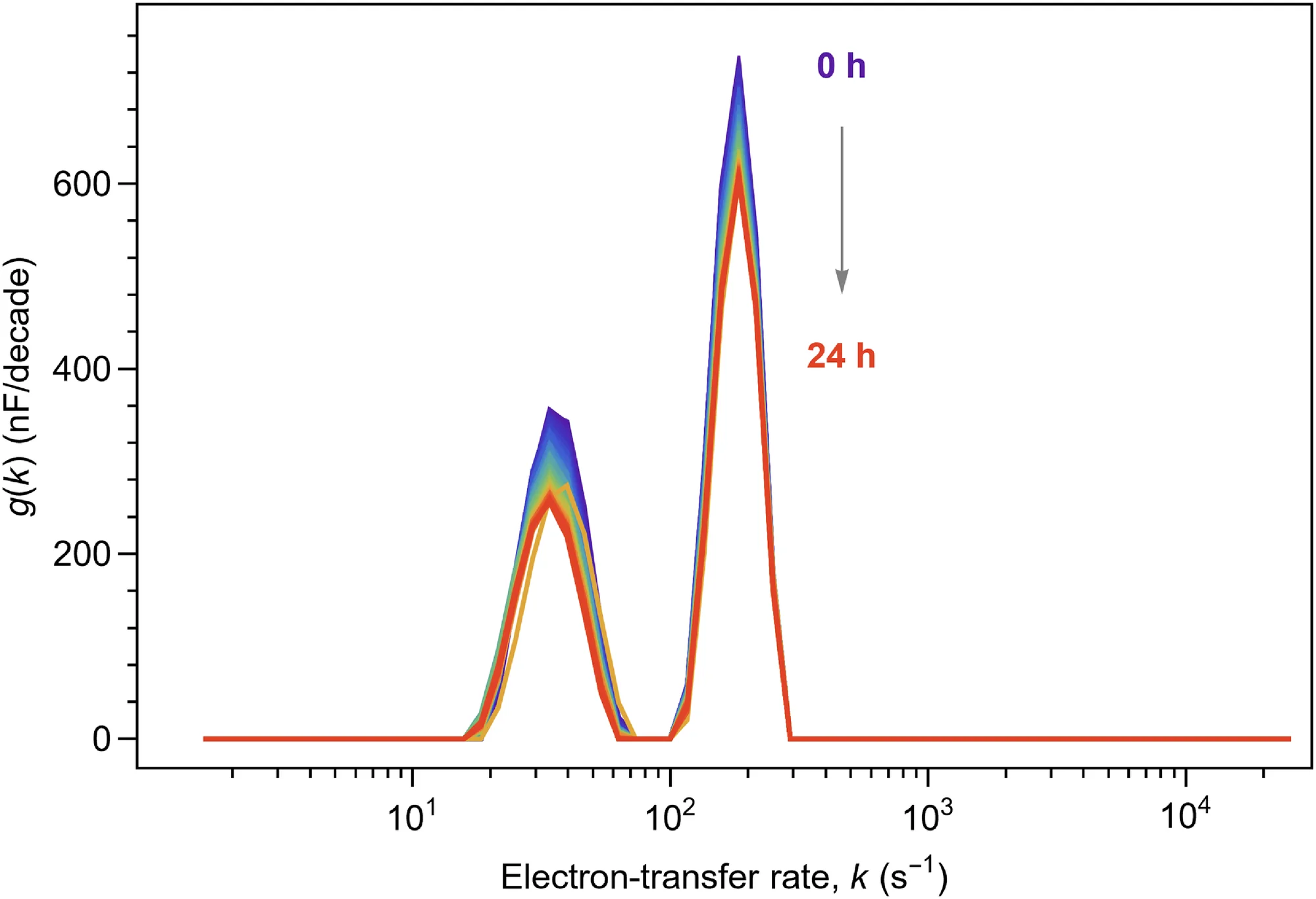
The two well-separated peaks here are the central observation enabling this work: a vancomycin EAB sensor has two discrete electron-transfer populations, not a single broad distribution. DCT spectra shown were acquired on Electrode B1 over 24 h at 1 mM vancomycin (1 × PBS, 0.7 mM MgCl_2_, 37 °C); both peaks decline in amplitude over time (blue → red), consistent with gradual loss of surface-bound aptamers.

Having established to our satisfaction that the two peaks are robust and reflect the electron transfer kinetics of the vancomycin sensor, we next examined the dependence of these two peaks on analyte concentration. To do so, we varied the vancomycin concentration from zero to 1 mM, measured the EIS spectrum at each concentration (Figs. 4a and 4b), and performed DCT analysis (Fig. 4c). We normalized the raw DCT spectra *g*(*k*) by the total area under each curve, *C*_dist_, to obtain normalized DCT spectra (Figs. 5 and S14). Such normalization facilitates a comparison between the relative occupancy of the two states even as the total number of aptamers declines over time due to surface loss.^39^ In doing this, we find that, while their electron transfer kinetics remain approximately constant (Fig. S15), the occupancy of each of the two states changes with concentration: as concentration increases, a larger fraction of the molecules occupy the more rapid electron-transfer-rate state.

**Figure 4.**
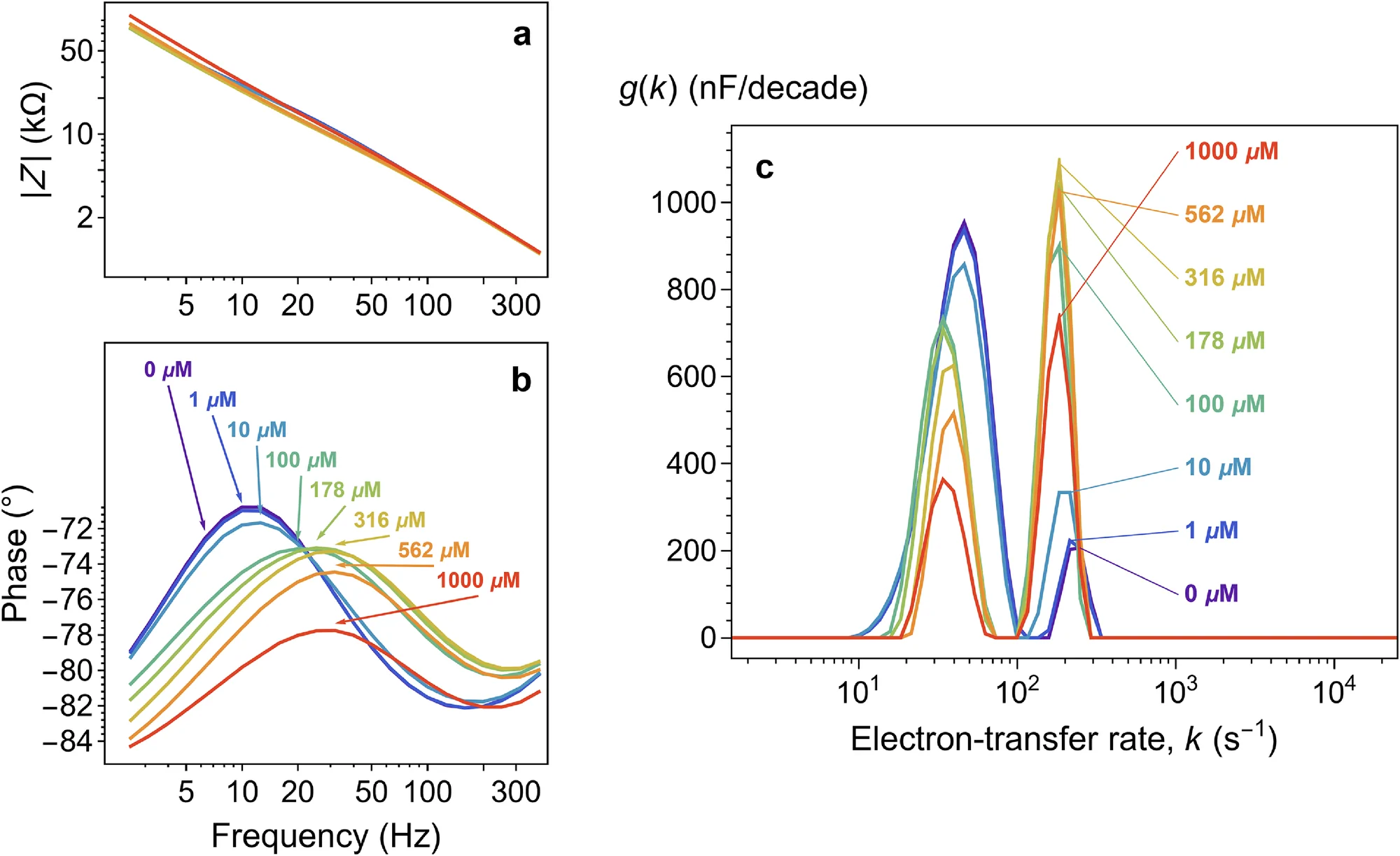
Target binding redistributes the aptamers of a vancomycin EAB sensor between two states rather than continuously shifting one population’s electron-transfer rate. A single peak in the raw EIS phase data, which shifts to higher frequency as concentration increases (a, b show Bode magnitude and phase), is resolved by DCT (c) into two peaks at nearly constant rates whose relative occupancy shifts toward the faster state as concentration increases from 0 to 1000 µM. The total peak area decreases with increasing concentration due to ongoing aptamer loss (appendix, Eq. A.2). Data shown is from Electrode B1.

**Figure 5.**
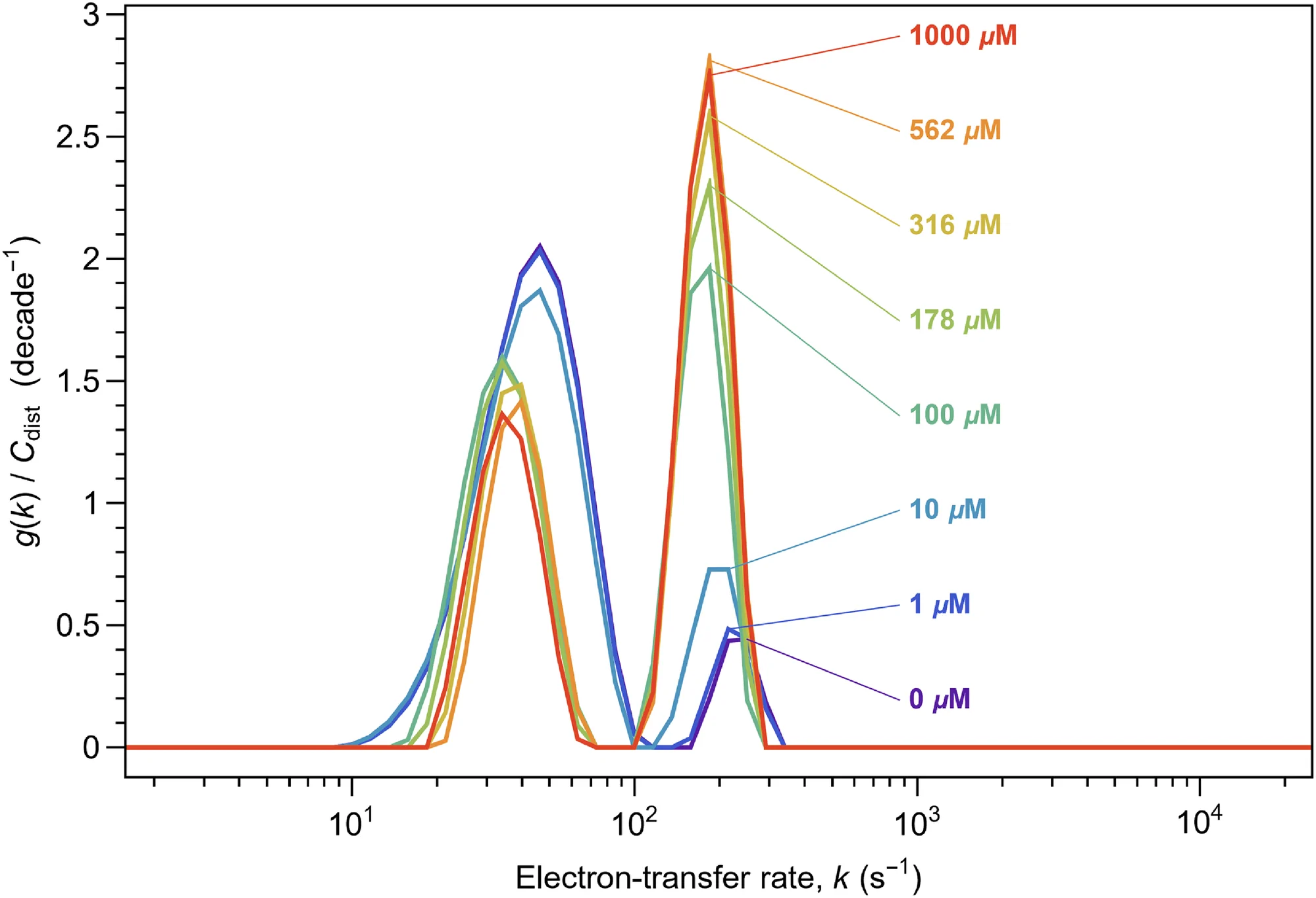
As vancomycin concentration increases, aptamers move from the low-electron-transfer-rate state to the high-electron-transfer-rate state while the rates themselves stay relatively constant. Here, each spectrum is normalized to its own total area (*C*_dist_) so that the population shift is readily apparent without the confounding effect of aptamer loss over time. Data shown is from Electrode B1.

### Analysis of fast-mode capacitive fraction versus concentration to probe biophysical models.

We further explore the dependence of the fast-mode capacitive fraction on concentration by fitting it to a biophysical model to advance our understanding of the system biophysics. Specifically, we numerically integrated the area under the curve between 10 s^−1^ and 100 s^−1^ to determine the capacitance contribution of the slow mode ${C_\mathrm{s}}$, and between 100 s^−1^ and 500 s^−1^ to determine the capacitance contribution of the fast mode ${C_\mathrm{f}}$. With additional assumptions, this faradaic capacitance is proportional to number of aptamers in each mode (appendix). We can calculate the fast-mode capacitive fraction as ${f_\mathrm{f}} = \frac{{C_\mathrm{f}}}{{C_\mathrm{f}}+{C_\mathrm{s}}}$, which is plotted as a function of concentration (Fig. 6) and as a function of time (Fig. S16). Previous work introduced a three-state model for EAB sensors,^5^ in which a non-binding, binding-competent, and bound state are used to describe a DNA aptamer and its binding to a target sequence. Here, we adapt the same model to our experiment, assuming that the relevant states are the unfolded aptamer (*U*), its folded, binding-competent, but unbound state (*F*), and the folded, target-bound complex (*B*). We further assume that these underlying molecular states map onto the electrochemically measurable fast-and slow-modes as: [F*] = [*F*] + [*B*] and [S*] = [*U*], with square bracket notation to indicate occupancy of each state. In order to make an analytical equation that predicts the occupancy of the states, we make three additional assumptions. First, we assume that the relative occupancy of the folded state is given by a switching equilibrium constant *K_S_* = [*F*]/[*U*]. Second, we assume that the relative occupancy of the bound state as a function of target concentration *C*_target_ is given by the intrinsic affinity *K_D_*^int^ of the folded, binding-competent state such that: [*B*] = [*F*] *C*_target_/(*K_D_*^int^). Third, we assume that the sum of all the state occupancies must sum to unity: [*U*] + [*F*] + [*B*] = 1. We can then solve the system of equations to arrive at a biophysical model which provides the fast-mode capacitive fraction as a function of concentration and depends on two model-dependent parameters and one experimental variable:

\begin{equation*}{f_\mathrm{f}} = \frac{\left[ F\ast \right]}{\left[ F\ast \right]+\left[ S\ast \right]} = \frac{\left( {C_{\mathrm{target}}}+{{K_D}^{\mathrm{int}}} \right){K_S}}{{{K_D}^{\mathrm{int}}}+\left( {C_{\mathrm{target}}}+{{K_D}^{\mathrm{int}}} \right){K_S}}\end{equation*}

**Figure 6.**
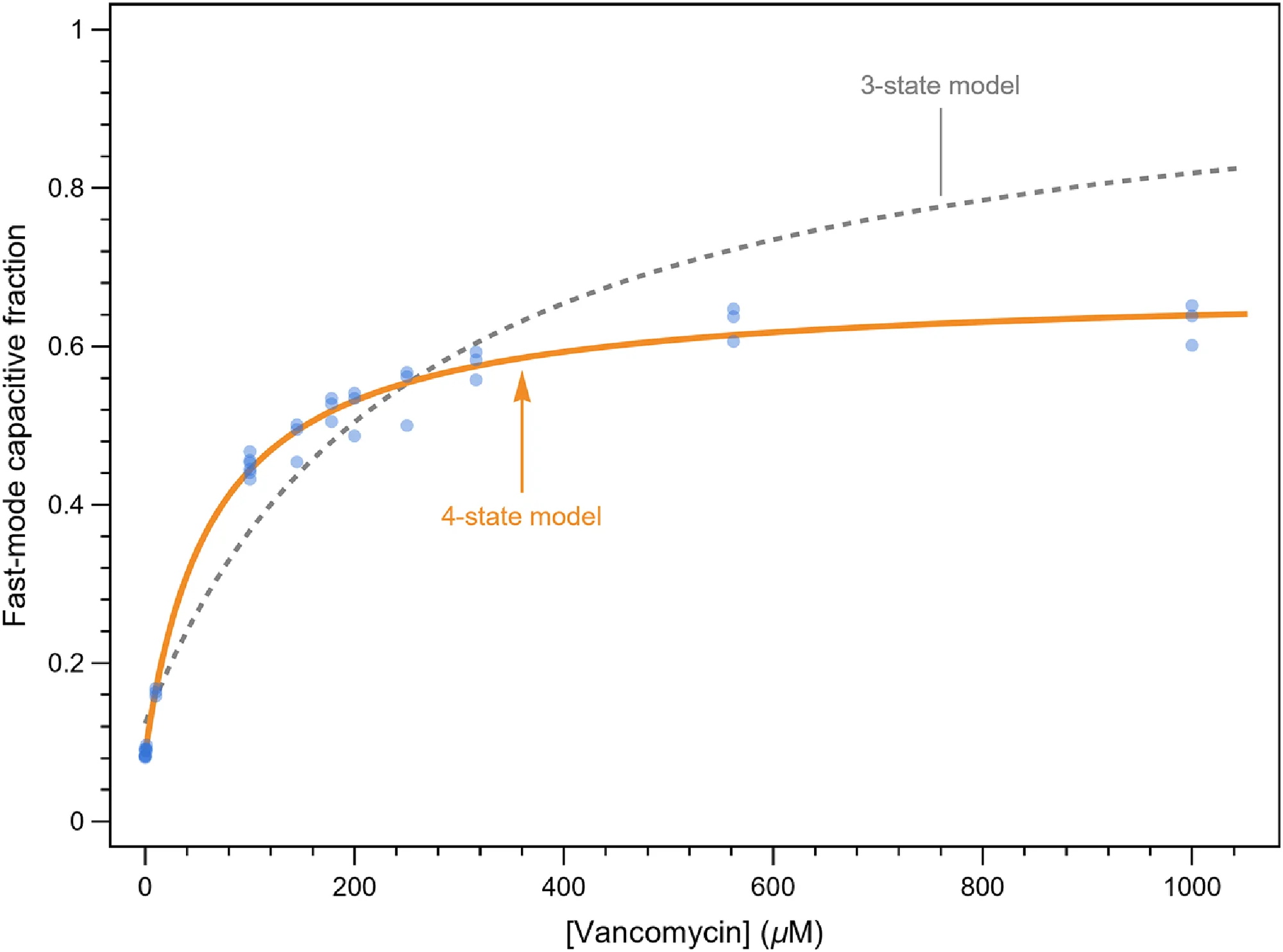
The fast-mode capacitive fraction of a vancomycin EAB sensor (blue dots), plotted against target concentration, plateaus near 60% rather than 100%. The three-state model (grey dashed line) cannot reproduce both the low-and high-concentration behavior (*R*^2^ = 0.974, RMSD = 0.073), whereas a four-state model that includes a non-folding population (orange line) captures the full titration (*R*^2^ = 0.998, RMSD = 0.018), consistent with a model in which roughly a third of surface aptamers are non-folding and never respond to target. This plot combines the data from all six electrodes: A1, A2, A3, B1, B2, B3.

When we fitted this 3-state model (Eq. 9) to our experimental data, however, we observe a significant, systematic offset (Fig. 6). Specifically, rather than asymptotically approaching 100% as vancomycin concentrations rise, the experimental data instead asymptotes at only ∼60%. A potential explanation for this is that not all of the surface-bound aptamers are capable of folding (and thus do not respond to target), perhaps due to close packing with neighbors, to being located in crevices within the rough gold used in these experiments, to sequence errors in the DNA synthesis, or to their being adsorbed onto the gold surface. To correct for the presence of “folding incompetent” aptamers, we add an additional assumption and introduce a fourth, “non-folding” state (non-folding, NF, Fig. 7) rewriting [S*] = [*U*] + [NF], and [*U*] + [*F*] + [*B*] + [NF] = 1, to obtain:

\begin{equation*}{f_\mathrm{f}} = \frac{\left[ F\ast \right]}{\left[ F\ast \right]+\left[ S\ast \right]} = \frac{\left( {C_{\mathrm{target}}}+{{K_D}^{\mathrm{int}}} \right){K_S}\left( 1-\left[ \mathrm{NF} \right] \right)}{{{K_D}^{\mathrm{int}}}+\left( {C_{\mathrm{target}}}+{{K_D}^{\mathrm{int}}} \right){K_S}}\end{equation*}

**Figure 7.**
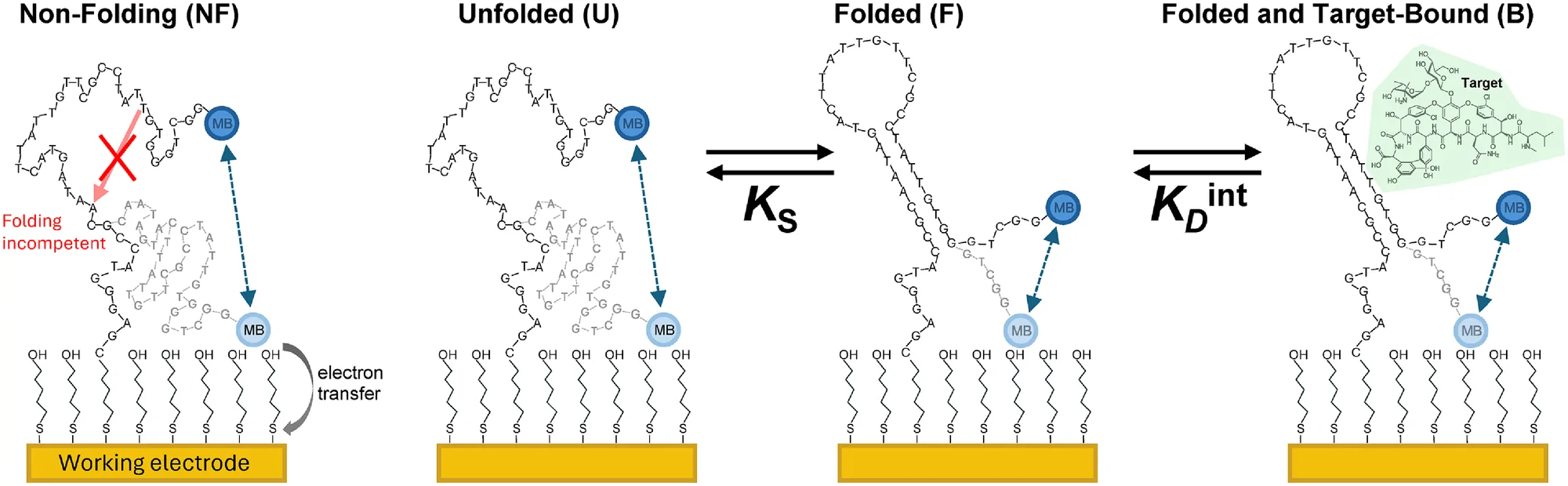
A proposed four-state model, in which aptamers exchange between non-folding (NF), unfolded (*U*), folded (*F*), and target-bound (*B*) states. The NF population is permanently unable to fold, while the *U, F*, and *B* states exchange according to their equilibrium constants. A candidate structure for the folded state was generated from the 4-trunc aptamer sequence^22^ using UNAFold.^47^

We find that this 4-state model (Eq. 10) has excellent agreement with the measured fast-mode capacitive fraction across all six electrodes and concentrations tested (Fig. 6), reducing the root-mean-square deviation (RMSD) from 0.073 for the three-state model to 0.018, and yielding the following model-dependent estimates: *K*_*S*_ = 0.15, *K_D_*^int^ = 8.5 μM, [NF] = 0.32. That latter value provides a model-dependent estimate of 32% for the fraction of aptamers on the surface of this sensor that are incapable of folding in response to their target.

## Results and Discussion

Applying DCT to EAB sensors uncovers two electron-transfer-rate populations where previous methods saw only one. Prior square-wave voltammetry and Lovrić-plot analysis of this sensor showed that target binding shifts the apparent electron-transfer rate in a comparable frequency range,^26^ but lacked the resolution to reveal its origin; DCT resolves this shift into the transfer of population between two states with characteristic rates of ∼35 s^−1^ and ∼180 s^−1^. Prior work suggested that the target-bound and target-free states of the EAB sensor’s aptamer would necessarily create distinct peaks in the device’s EIS phase response.^42^ We show instead that a broad single peak in the experimental Bode phase plot of a vancomycin EAB sensor, spanning 5–100 Hz (Fig. 4b), can be resolved into two distinct electron transfer rate peaks using DCT (Fig. 4c). Our finding of a clearly resolved slow-mode and a fast-mode of electron transfer rate contrasts with previous attempts to fit EIS data collected from EAB sensors using a single Constant Phase Element and resistor in parallel to the double layer capacitance,^7^^,^^43^ which, since these models are incompatible with a dual-peaked electron-transfer-rate distribution, are an oversimplification.

In order to test how robust our results are to changes in the model parameters we performed a sensitivity analysis (Table S2). We find that the presence of two distinguishable peaks is robust across the range of parameters tested. However, the values of the concentration-dependent electron-transfer-rates and biophysical-model fits, which relied on the concentration dependence of the two peaks, were more sensitive. For example, changing *f*_max_ from 500 Hz to 628 Hz resulted in the electron transfer rate increasing rather than decreasing with concentration (Fig. S17), so although it appears as if the mean weighted electron transfer rates of the two peaks are slightly decreasing with concentration, this may be a numerical artifact. Interestingly, changing the assumed weighting parameter from *P* = 0.87 (our best fit) to 0 (uniformly weighted data) resulted in fast-mode capacitive fraction that asymptotes to nearly one (Fig. S18). However, we assume that our weighting scheme is reasonable and that the four-state model we proposed is consistent with the data. Furthermore, electrochemical measurements alone cannot teach us whether the observed slow-mode (S*) and fast-mode (F*) correspond to reporter orientation, the surface-binding environment, formal potential spread, or nonspecific adsorption,^43^ although our observations are consistent with a biophysical model wherein the fast-mode corresponds to the folded states and the slow-mode corresponds to the unfolded states. Our biophysical model analysis also assumes that the two electrochemical modes share comparable electrochemical visibility, formal-potential dependence, and pseudocapacitance per molecule at the measurement potential. Additional measurements to challenge these assumptions are warranted.

The work described here provides a number of new insights into the mechanisms underlying EAB sensors. For example, previous work demonstrated increased signal gain upon codeposition of the aptamer with the target,^44^ an effect that was hypothesized to reduce the number of deposited aptamers that are incapable of folding (NF). DCT now provides a model-dependent estimate of this fraction. DCT similarly provides a means of addressing another question recently posed in the EAB sensor community: is the binding-induced change in average electron transfer rate due to structure (i.e. conformational) switching, as described here, or due to a binding-induced change in the reorganization energy of the redox reporter caused by a binding induced change to the local environment?^38^ In the conformational-switching model, the aptamer populates a dynamic equilibrium in which it interconverts between a low-electron-transfer-rate state (unfolded) and a high-electron-transfer-rate state (folded) even in the absence of target. Binding to the folded state then shifts the equilibrium toward the folded state. In the reorganization-energy model, by contrast, formation of the high-electron-transfer rate state is directly induced by binding. Because of that, no mechanism exists in the reorganization-energy model to populate the high transfer state in the absence of target. Using DCT combined with a biophysical model, however, we find that, at least for the vancomycin sensor employed here, approximately 9% of the aptamers occupy the high electron-transfer rate state even in the absence of target. This is more consistent with the binding-induced conformational-change model than the reorganization-energy model for this particular sensor. However, since the net electron transfer rate of a state is: ${k_{\mathrm{state}}} = \int_0^\infty P(z)k(z)\mathrm{dz}$, where k(z) is the electron transfer rate as a function of distance, typically an exponentially decaying function of distance that also depends on reorganization energy, and P(z) is the probability of the redox reporter being a given distance from the surface, our work still leaves an open question: are there two resolvable electrochemical modes because the aptamer adopts two conformations, each with equivalent *k*(*z*) distributions (our schematic in Fig. 7), or does vancomycin binding induce a reorganizational energy change that happens to match the reorganizational energy of the naturally fluctuating fast state? The former is our preferred explanation and is consistent with the data, since the latter relies on a coincidental alignment of the reorganizational energy between a fluctuating aptamer with no target present into a faster state and that of a target-bound aptamer. Whether our finding of binding-and-switching over binding also holds for other aptamers employed in other EAB sensors remains an open question, one of many^45^ that we believe DCT is well-poised to answer, including: “is a dual-peaked Cole-Cole distributed electron transfer rate model a good parameterization of our dual-peaked DCT results?”^43^ and “can DCT resolve the influence of nanostructuring on electron transfer in EAB sensors?”^46^ Beyond EAB sensors, we anticipate that DCT could also be applied to other surface-immobilized redox reactions to improve understanding of the magnitude and sources of their heterogeneity.

Although both our work and the recent study by Martinez-Blanco et al.^48^ apply DCT to related electrochemical systems, there are several notable differences. First, whereas they fit their EIS data to a Maxwell ladder, we took the additional step of accounting for series solution resistance first, which enables our results to provide interfacial DCT spectra rather than system DCT spectra, facilitating comparison across different experimental conditions where solution resistance may vary. When we fit our data with only a parallel resistor, we found that the two peaks were not well-resolved (Fig. S19), suggesting that accounting for solution resistance before performing DCT analysis is a critically important step. Secondly, whereas they measured aptamer response to a complementary DNA sequence which bound to it, we measured a vancomycin EAB sensor and added vancomycin. As a result, they found that it was difficult to interpret the influence of cDNA on their resulting spectra, which their prior work attributed to adsorption,^43^ while we found that a vancomycin EAB sensor is well-described by a four-state binding and folding model, with two clearly resolvable peaks that are relatively stable in their respective electron transfer rates while varying in occupancy with vancomycin concentration. Third, they used Gaussian processes, which if the underlying assumptions are true, can yield more accurate results, whereas we used a linear algebra based approach and a sensitivity analysis on our fit parameters. Finally, they used a 4th electrode with a shunt capacitor (10 nF) to compensate for any stray capacitance due to the salt bridge, whereas we did not. Future work could explore whether our lack of shunt capacitor could perhaps explain the dip in variance we observe around 1000 Hz (Fig. S4).

Although DRT and DCT are two possible kernel functions, DRT being the most commonly used, and DCT finding applicability in this work, there are infinitely many possible kernel functions to choose from. We agree with Orazem and Ulgut,^12^ who state that DRT is not model-free and that claims that DRT is model-free^21^^,^^48^^–^^51^ are incorrect. However, we believe that this work, highlighting an experimental system where the DCT kernel (Eq. 1) has excellent agreement with the physics of a surface-bound redox probe system, provides a stepping stone for future distribution of timescales methods beyond DRT and DCT. It is exciting to consider which kernels could apply to which physical systems in fields such as molecular sensors, electrocatalysts, corrosion layers, batteries, and composite electrodes, and where other physics plays an important role such as diffusion. For example, the Distribution of Diffusion timescales kernel^52^ may have broader applicability than has been appreciated thus far in the literature.

## Conclusions

When using a DRT, DCT, or any distribution-of-timescales approach, it is essential that the assumed kernel matches the physical system. We find excellent agreement between the EAB sensor and the Maxwell kernel of DCT over a broad frequency range. We use DCT to analyze vancomycin EAB sensor data and after removing the series solution resistance, find that there are two clearly resolvable electron transfer rate peaks, a fast-mode and a slow-mode. We measure the fast-mode capacitive fraction as a function of concentration, and find that the results can be fit by adapting a previously published three-state biophysical model and adding a fourth non-folding state. The DCT method we describe here will enable advances in the characterization of heterogeneous electrochemical surfaces in a wide variety of applications. Our research paves the way for future theorists to derive new kernels to describe physical systems and for experimentalists to carefully choose kernels to match their system of interest.

## Appendix Relating DCT spectra to surface density

Complementary measurements can also help us to relate the measured DCT spectra *g*(*k*) (capacitance per decade), to surface density Γ (mol cm^−2^). Near the formal potential $E^{0^{\prime}}$, and at frequencies much lower than the electron transfer rates, the reduced fraction θ follows from the Nernst equation:

\begin{equation*}\theta = \frac 1{1+{e^{\frac{\mathrm{nF}}{\mathrm{RT}}\left(E-E^{0^{\prime}}\right)}}}\end{equation*}

where *E* is the applied potential, *E*^0^′ the formal potential, *n* the number of electrons, and *F, R, T* have their usual meanings. With a surface charge of $Q = n~F~A~\Gamma ~\theta ,$ and taking the derivative of charge with respect to applied potential E about the formal potential $E^{0^{\prime}}$, we obtain the pseudo-capacitance:

\begin{equation*}{C_{p,\max}} = \frac{\mathrm{d} Q}{\mathrm{d} E} = \frac{{n^2}{F^2}A~\Gamma}{4\mathrm{RT}}.\end{equation*}

In the case of an electrochemical aptamer-based biosensor, which utilizes surface-immobilized methylene blue, with *n =* 2 electrons per molecule, with a cylindrical electrode with a geometric surface area of $A = \pi \mathrm{Dh} = \pi \times 0.2~\mathrm{mm}\times 3~\mathrm{mm} = 1.9\times {10^{-2}}{\mathrm{cm}^2}$, and a measured typical total faradaic capacitance obtained from DCT of ${C_{p,\max}} = \int g(k)\mathrm{d} ({\log_{10}}\mathrm{k} )$ = 250 nF, we can rearrange Eq. A.2 to obtain the surface density:

\begin{align*}\Gamma &amp;= \frac{C_{p,\max}\,4\mathrm{RT}}{n^{2}F^{2}A} \nonumber\\ &amp;= \frac{\left(2.50\times10^{-7}\ \mathrm{A\,s\,V^{-1}}\right)\,4\left(8.314\ \mathrm{J\,mol^{-1}\,K^{-1}}\right)\left(310\ \mathrm{K}\right)}{2^{2}\left(96485\ \mathrm{A\,s\,mol^{-1}}\right)^{2}\left(1.9\times10^{-2}\ \mathrm{cm^{2}}\right)} \nonumber\\ &amp;= 3.6\ \mathrm{pmol\,cm^{-2}}.\end{align*}

## References

[1] Bard A. J., Faulkner L. R., White H. S. (2022). Electrochemical Methods: Fundamentals and Applications.

[2] Eckermann A. L., Feld D. J., Shaw J. A., Meade T. J. (2010). Coord. Chem. Rev..

[3] Dehnari E., Jamshidi Z., Taherinia D. (2025). Electrochim. Acta.

[4] Smalley J. F., Finklea H. O., Chidsey C. E. D., Linford M. R., Creager S. E., Ferraris J. P., Chalfant K., Zawodzinsk T., Feldberg S. W., Newton M. D. (2003). J. Am. Chem. Soc..

[5] Vallée-Bélisle A., Ricci F., Plaxco K. W. (2009). Proc. Natl Acad. Sci. USA.

[6] Chang B.-Y., Park S.-M. (2010). Annu. Rev. Anal. Chem..

[7] Roehrich B., Leung K. K., Gerson J., Kippin T. E., Plaxco K. W., Sepunaru L. (2023). ACS Sens..

[8] Corcoran N., Dauphin-Ducharme P. (2026). J. Electrochem. Soc..

[9] Xia J., Wang C., Wang X., Bi L., Zhang Y. (2020). Electrochim. Acta.

[10] Reshetenko T., Kulikovsky A. (2020). J. Electrochem. Soc..

[11] Maradesa A. (2024). Joule.

[12] Orazem M. E., Ulgut B. (2025). J. Electrochem. Soc..

[13] Py B., Maradesa A., Ciucci F. (2024). Electrochim. Acta.

[14] Schönleber M., Ivers-Tiffée E. (2015). Electrochem. Commun..

[15] Py B., Wang F., Liu J., Wang X., Wang Z., Wu P. M., Wu M.-K., Wang J., Ciucci F. (2026). J. Electrochem. Soc..

[16] Allagui A., Majzoub S., Elwakil A. (2026). https://arxiv.org/abs/2606.17066.

[17] Arroyo-Currás N., Scida K., Ploense K. L., Kippin T. E., Plaxco K. W. (2017). Anal. Chem..

[18] Buteau S., Dahn D. C., Dahn J. R. (2018). J. Electrochem. Soc..

[19] Tikhonov A. N. (1963). Dokl. Akad. Nauk SSSR.

[20] Lawson C. L., Hanson R. J. (1995). Solving Least Squares Problems.

[21] Hahn M., Schindler S., Triebs L.-C., Danzer M. A. (2019). Batteries.

[22] Shaver A., Mahlum J. D., Scida K., Johnston M. L., Pellitero M. A., Wu Y., Carr G. V., Arroyo-Currás N. (2022). ACS Sens..

[23] Fan C., Plaxco K. W., Heeger A. J. (2003). Proc. Natl Acad. Sci. USA.

[24] Ferguson B. S. (2013). Sci. Transl. Med..

[25] Arroyo-Currás N., Somerson J., Vieira P. A., Ploense K. L., Kippin T. E., Plaxco K. W. (2017). Proc. Natl Acad. Sci. USA.

[26] Dauphin-Ducharme P., Yang K., Arroyo-Currás N., Ploense K. L., Zhang Y., Gerson J., Kurnik M., Kippin T. E., Stojanovic M. N., Plaxco K. W. (2019). ACS Sens..

[27] Arroyo-Currás N., Dauphin-Ducharme P., Scida K., Chávez J. L. (2020). Anal. Methods.

[28] Idili A., Gerson J., Kippin T., Plaxco K. W. (2021). Anal. Chem..

[29] Chamorro-Garcia A. (2023). ACS Sens..

[30] Abeykoon S. W., White R. J. (2024). Anal. Chem..

[31] Verrinder E., Gerson J., Leung K., Kippin T. E., Plaxco K. W. (2024). ACS Sens..

[32] Verrinder E., Leung K. K., Erdal M. K., Sepunaru L., Plaxco K. W. (2024). Sens. Diagn..

[33] Clark V., Prasad A., Yuan Y., Demek E., Guzman F., Garcia L. A., Lukeman P. S., Powell-Gray B., Rodríguez-López J., Arroyo-Currás N. (2025). ACS Sens..

[34] Zhu M., Xie C., Xu F., Li S., Li H., Xia F. (2025). ACS Sens..

[35] Son K., Gibson J. M., Gerson J., Leung K. K., Stocco M., Lu Y., Kippin T. E., Plaxco K. W. (2026). J. Am. Chem. Soc..

[36] Booth M. A. (2026). Nat. Biotechnol..

[37] Dauphin-Ducharme P., Dief E. M., Corcoran N., Gooding J. J. (2025). ACS Electrochem..

[38] Deflandre L., Dauphin-Ducharme P. (2024). ACS Sens..

[39] Leung K. K., Downs A. M., Ortega G., Kurnik M., Plaxco K. W. (2021). ACS Sens..

[40] Leung K. K., Gerson J., Emmons N., Heemstra J. M., Kippin T. E., Plaxco K. W. (2024). Angew. Chem., Int. Ed..

[41] Pellitero M. A., Curtis S. D., Arroyo-Currás N. (2021). ACS Sens..

[42] Dauphin‐Ducharme P., Churcher Z. R., Shoara A. A., Rahbarimehr E., Slavkovic S., Fontaine N., Boisvert O., Johnson P. E. (2023). Chem. Eur. J..

[43] Ma T., Baker D. V., Martinez-Blanco G., Bizzotto D. (2025). Electrochim. Acta.

[44] Wu Y., Shi J., Kippin T. E., Plaxco K. W. (2024). Langmuir.

[45] Rahbarimehr E., Chao H. P., Churcher Z. R., Slavkovic S., Kaiyum Y. A., Johnson P. E., Dauphin-Ducharme P. (2023). Anal. Chem..

[46] Huldin G. F., Huang J., Fu K. X. (2025). Curr. Opin. Electrochem..

[47] Markham N. R., Zuker M., Keith J. M. (2008). Bioinformatics.

[48] Martinez-Blanco G., Baker D. V., Py B., Rassachack E., Ciucci F., Bizzotto D. (2026). Electrochim. Acta.

[49] Schichlein H., Müller A. C., Voigts M., Krügel A., Ivers-Tiffée E. (2002). J. Appl. Electrochem..

[50] Danzer M. A. (2019). Batteries.

[51] Zhu D., Ma T., Yang Y. (2022). J. Power Sources.

[52] Song J., Bazant M. Z. (2018). Phys. Rev. Lett..

